# Quantifying effects of stochasticity in reference frame transformations on posterior distributions

**DOI:** 10.3389/fncom.2015.00082

**Published:** 2015-07-03

**Authors:** Hooman Alikhanian, Schubert R. de Carvalho, Gunnar Blohm

**Affiliations:** ^1^Centre for Neuroscience Studies, Queen's UniversityKingston, ON, Canada; ^2^Canadian Action and Perception NetworkKingston, ON, Canada; ^3^Association for Canadian Neuroinformatics and Computational NeuroscienceKingston, ON, Canada

**Keywords:** reaching, sensory-motor transformation, reference frame transformation, Stochastic noise, deviation from normality

## Abstract

Reference frame transformations are usually considered to be deterministic. However, translations, scaling or rotation angles could be stochastic. Indeed, variability of these entities often originates from noisy estimation processes. The impact of transformation noise on the statistics of the transformed signals is unknown and a quantification of these effects is the goal of this study. We first quantify analytically and numerically how stochastic reference frame transformations (SRFT) alter the posterior distribution of the transformed signals. We then propose an new empirical measure to quantify deviations from a given distribution when only limited data is available. We apply this empirical measure to an example in sensory-motor neuroscience to quantify how different head roll angles change the distribution of reach endpoints away from the normal distribution.

## 1. Introduction

Reference frame transformations are crucial components in many areas of science and technology. This includes Engineering, Computer Graphics, Physics, Robotics, Mathematics, and Neuroscience. Until now they have been used in a deterministic fashion, i.e., assuming that we have exact knowledge about the transformation parameter, such as rotational angles and axes. However, in real world applications these transformation parameters are often noisy estimates. For example, measurement errors can result in noisy parameter estimates. Here, we are interested in describing the impact of noise in reference frame transformations on the statistical distribution of transformed data. We propose that reference frame transformations should sometimes more appropriately be described in stochastic terms, i.e., stochastic reference frame transformations (SRFTs), and demonstrate the impact of SRFTs for Neuroscience research, but our findings generalize to other areas.

In Neuroscience—our application field of choice—reference frame transformations are omnipresent (Knudsen et al., 1987; Soechting and Flanders, 1992; Lacquaniti and Caminiti, 1998; Snyder, 2000; Cohen and Andersen, 2002; Engel et al., 2002; Henriques et al., 2002; Crawford et al., 2004; Buneo and Andersen, 2006; Schlicht and Schrater, 2007; Tagliabue and McIntyre, 2014) and we therefore expect a large impact of noise on transformed data and thus the neuronal and behavioral outcomes. For example sensory signals enter the brain in different frames of reference (vision in a retinal frame, audition in a head-centered frame, etc) and drive different motor systems (e.g., eye, head, arm movement) requiring motor commands to be specified in yet again different coordinate frames. Thus virtually all sensory-motor computations are affected by the inevitable stochasticity of reference frame transformations (Rossetti et al., 1994; Sober and Sabes, 2003, 2005; Blohm and Crawford, 2007; Schlicht and Schrater, 2007; McGuire and Sabes, 2009; Burns and Blohm, 2010; Burns et al., 2011).

In the present manuscript, we provide new methods for quantifying SRFTs. First, we compute the exact change in the statistical posterior distribution compared to the original distribution due to SRFTs. In a second step, we propose a new measure to quantify deviations of statistical distributions from their original distribution in limited experimental data. Finally, we validate our hypotheses and approach on previously published data from a reaching task performed under different head roll positions (Burns and Blohm, 2010) to demonstrate that larger reference frame transformations will lead to a larger deviations from normality. Together, these three steps are the building blocks for capturing the effects of SRFTs on experimental data.

## 2. Methods

### 2.1. SRFT analysis

We will consider the following general linear transformation of original data points P_0_ into corresponding data P_1_.

(1) $${\text{P}}_{1}\;=\;R{\text{P}}_{0}$$

Our first goal was to find the distribution of the transformed data P_1_ given P_0_ with a known distribution and a noisy transformation matrix **R** = **R**(*k*, θ), where *k* is a scaling factor and θ is a rotation angle. Without loss of generality, we will use two-dimensional (2D) data and transformations of the form:

(2) $$R=k\left(\begin{array}{cc} \cos\theta & -\sin\theta \\ \sin\theta & \cos\theta \end{array}\right)$$

,and investigate the effect of noise in both *k* and θ on the resulting distribution of *P*_1_. (Note that the linear transformation depends non-linearly on θ and is thus expected to result in non-linear effects). To this end, we assume that the data to be transformed is normally distributed because normality is assumed in almost all behavioral neuroscience data, but our conclusions and mathematical developments also apply for other distributions. Because rotation is a linear transformation, the transformed data should be jointly normal as well. Therefore, it is possible to investigate the effect of noisy transformations by measuring the amount of deviation from normality on the transformed data.

We assume that the data vector **P**_0_ = (*x*_1_, *x*_2_) contains two jointly normal random variables with variances σ^2^_*x*_*i*__, *i* = {1, 2}, and correlation coefficient ρ with the following joint distribution:

(3) $$\begin{array}{l} f_{X}(x_{1},x_{2})=\frac{1}{2\pi \sigma_{x_{1}}\sigma_{x_{2}}\sqrt{1-\rho^{2}}}exp(-\frac{1}{2(1-\rho^{2})}\\ \{\frac{{\left(x_{1}-\mu_{1x}\right)}^{2}}{\sigma_{x_{1}}^{2}}+\frac{{\left(x_{2}-\mu_{2x}\right)}^{2}}{\sigma_{x_{2}}^{2}}-\frac{2\rho (x_{1}-\mu_{x_{1}})(x_{2}-\mu_{x_{2}})}{\sigma_{x_{1}}\sigma_{x_{2}}}\}). \end{array}$$

Let us further assume that the data is going to be transformed by the transformation matrix **R** to the new vector **P**_1_ = (*y*_1_, *y*_2_) = **y**. The goal is then to find the distribution of the resulting transformed vector **y** when the angle of rotation θ, and the scaling factor *k* are noisy with known distributions *f*_θ_(θ), and *f*_*k*_(*k*), respectively. If we assume that the data is jointly normal, then the resulting linearly transformed vector **y** would be jointly normal as well with the following distribution:

(4) $$\begin{array}{l} f_{Y}(y_{1},y_{2}|\theta ,k)=\frac{1}{2\pi \sigma_{y_{1}}\sigma_{y_{2}}\sqrt{1-\rho_{y}^{2}}}exp(-\frac{1}{2(1-\rho_{y}^{2})}\\ \{\frac{{\left(y_{1}-\mu_{1y}\right)}^{2}}{\sigma_{y_{1}}^{2}}\;+\;\frac{{\left(y_{2}-\mu_{2y}\right)}^{2}}{\sigma_{y_{2}}^{2}}-\frac{2\rho_{y}(y_{1}-\mu_{y_{1}})(y_{2}-\mu_{y_{2}})}{\sigma_{y_{1}}\sigma_{y_{2}}}\}), \end{array}$$

where μ_*y*_1__, μ_*y*_2__, σ^2^_*y*_1__, σ^2^_*y*_2__, and ρ_*y*_ are defined, respectively as:

(5) $$\mu_{y_{1}}=k(\cos\theta \mu_{x_{1}}-\sin\theta \mu_{x_{2}}),$$

(6) $$\mu_{y_{2}}=k(\sin\theta \mu_{x_{1}}+\cos\theta \mu_{x_{2}}),$$

(7) $$\sigma_{y_{1}}^{2}=k^{2}(\cos^{2}\theta \sigma_{x_{1}}^{2}+\sin^{2}\theta \sigma_{x_{2}}^{2}-\rho \sin2\theta \sigma_{x_{1}}\sigma_{x_{2}}),$$

(8) $$\sigma_{y_{2}}^{2}=k^{2}(\sin^{2}\theta \sigma_{x_{1}}^{2}+\cos^{2}\theta \sigma_{x_{2}}^{2}+\rho \sin2\theta \sigma_{x_{1}}\sigma_{x_{2}}),$$

(9) $$\rho_{y}=\frac{\frac{1}{2}sin2\theta (\sigma_{x_{1}}^{2}-\sigma_{x_{2}}^{2})+\rho \sigma_{x_{1}}\sigma_{x_{2}}\cos2\theta }{\sigma_{y_{1}}\sigma_{y_{2}}}.$$

The distribution of the transformed vector **y** can now be defined as:

(10) $$f_{Y}(y_{1},y_{2})=\int_{k}\int_{\theta }f_{k}(k)f_{\theta }(\theta )f_{Y}(y_{1},y_{2}|\theta ,k)d\theta dk$$

Note that we have assumed that the scaling factor is independent of the angle of rotation. The integral in Equation (10) is difficult to be solved analytically. However, numerical estimation of the integral can provide us with a good understanding of the shape of the distribution of the transformed data **y**.

To quantify the influence of noise on the transformed data distribution, we calculated the distance of the resulting distribution from the distribution obtained with a noiseless transformation. Doing so, we can investigate the effect of noise in the rotation angle and scaling factor on the shape of the resulting distribution.

In this paper we use the Kullback–Leibler distance (Kullback and Leibler, 1951) to calculate the divergence of the two known distributions. Since the Kullback–Leibler distance is a non-symmetric measure, we specifically set out to quantify the information lost when using *f*_*Y,noiseless*_ (Equation 4) to approximate *f*_*Y*_ (Equation 10), such that:

(11) $$D_{KL}(f_{Y}||f_{Y,noiseless})=\int_{-\infty }^{\infty }f_{Y}\ln\frac{f_{Y}}{f_{Y,noiseless}}dx.$$

### 2.2. Assessing experimental deviations from multivariate normality

When the distribution of the transformed data is known or can be estimated numerically, it is possible to assess the deviation from multivariate normality (MVN) using the Kullback–Leibler distance (Equation 11). We use this in the first part of the results section for simulated data. However, in experiments, one typically does not have access to the noiseless distribution *f*_*Y,noiseless*_. Thus, to assess deviation from MVN for experimental data sets, two major groups of procedures have been used. The first group are statistical assessments that can test the hypothesis of data being normally distributed with a given *p*-value but such tests are not robust against sample size effects (Henderson, 2006). The second group of procedures uses graphical tools such as probability–probability plots (P–P) and quantile–quantile plots (Q–Q) (Wilk and Gnanadesikan, 1968; Thompson, 1990; Burdenski, 2000; Henderson, 2006).

Here, we propose a different approach to quantify the amount of deviation from normality that consists in (1) reducing the data space dimensionality and (2) estimating the empirical cumulative distribution function (CDF) of the transformed data samples and the reduced-dimension original data. We propose that the distance between the two empirical CDFs is a measure of deviation from normality.

In order to reduce the data space from 2D (or 3D) to 1D, we compute the sample's Mahalanobis distance:

(12) $${\text{d}}_{{\text{P}}_{\text{i}}}^{2}={\left({\text{P}}_{\text{i}}-\mu_{{\text{P}}_{0}}\right)}^{\text{T}}\Sigma_{{\text{P}}_{0}}^{-1}({\text{P}}_{\text{i}}-\mu_{{\text{P}}_{0}}),$$

where μ_*P*_0__ and Σ_*P*_0__ are the mean vector and covariance matrix of the original data (*P*_0_), respectively; 1 ≤ *i* ≤ *n* is the sample number. The Mahalanobis distance is computed for each sample of the transformed data *P*_1_.

The advantage of the Mahalanobis distance is twofold. It not only reduces the dimensionality of the data, but for MVN, the distance distribution also only depends on the dimensionality of the data, and does depend on neither the marginal standard deviations nor the correlation coefficients of data components. For MVN data with dimension *p*, the Mahalanobis distance distribution is χ^2^_p_ with *p* degrees of freedom. To study SRFTs, we thus compute the Mahalanobis distance for the data transformed without noise (*P*_1_) as well as the SRFT data (*P*_1_*SRFT*__), and estimate empirical CDFs for the two sample sets.

For independent identically distributed (IID) random variables *x*_*i*_, 1 ≤ *i* ≤ *n* with the common CDF *F*(*t*), the empirical CDF can be defined as:

(13) $$F_{x}^{emp}(t)=\frac{1}{n}\sum_{i=1}^{n}1\left(x_{i}\leq t\right),$$

where 1(.) is the indicator function.

Now the distance between the empirical CDFs of *d*^2^_*P*_1__ and *d*^2^_*P*_1_*SRFT*___ can be calculated as:

(14) $$D(F_{d_{P_{1}}^{2}}^{emp},F_{d_{P_{1_{SRFT}}}^{2}}^{emp})=\int_{0}^{1}{\left(F_{d_{P_{1}}^{2}}^{-1}(u)-F_{d_{P_{1_{SRFT}}}^{2}}^{-1}(u)\right)}^{2}du,$$

where *F*^−1^(.) is the inverse of the empirical CDF. The inverse exists because CDF is a monotonically increasing function for any random variable, and its domain is from 0 to 1.

To analyze the effects of noise in θ and *k*, we generated a random data sample, P_0_, and then applied transformations **R** with varying amounts of noise in θ and *k*.

### 2.3. Experimental proof of concept

Burns and Blohm (2010) showed that multi-sensory weights depend on contextual noise in reference frame transformation. They designed a reaching experiment to investigate the effect of head roll on sensory transformations and its consequences for multi-sensory integration weights. They showed that head orientation affects the weighting of visual and proprioceptive information in multi-sensory integration during reaching in two distinct ways. First, non-accurate head roll estimation results in an erroneous rotation of the visual information into proprioceptive coordinates. Second, non-reliable head roll estimation affects motor planning, and results in increased movement variability (Burns and Blohm, 2010). In other words, noise in the reference frame transformation between the rotated visual input during head roll and the spatially required movement resulted in more variable movements when the head was rolled as compared to when the head was straight. In this paper we use their data to show that reaching under head-roll conditions also results in deviations from normality compared to the head straight-ahead situation, confirming the hypothesis that head roll estimation noise underlies SRFTs of the visual information into proprioceptive coordinates.

Experimental procedures have been described in detail in Burns and Blohm (2010). Briefly, in their experiment they asked seven participants to perform a reaching task while seated in an augmented reality setup with their head position kept in place using a bite bar. Subjects viewed visual stimuli that were projected from an overhead screen through a semi-mirrored surface in six different positions at 10 cm distance from a center start position cross at 60, 90, 120, 240, 270, and 300° around the center cross. Underneath the mirrored surface, an opaque board prevented the subjects from viewing their hand. A dot corresponding to real time hand position provided subjects with feedback about their hand, but only until reach movements started at which time the hand position cue was removed. Subjects were instructed to begin each trial by aligning the visual cue representing their hand with the center cross. They performed rapid reaching movements using a vertical handle mounted on an air sled while keeping their gaze fixated on the center cross (Burns and Blohm, 2010). Participants completed the task at three different head roll positions, −30, 0, and 30° head roll. We used this data to analyze the distribution of reach directions compared to the normal distribution using Equation (14).

## 3. Results

**Figure 1:**
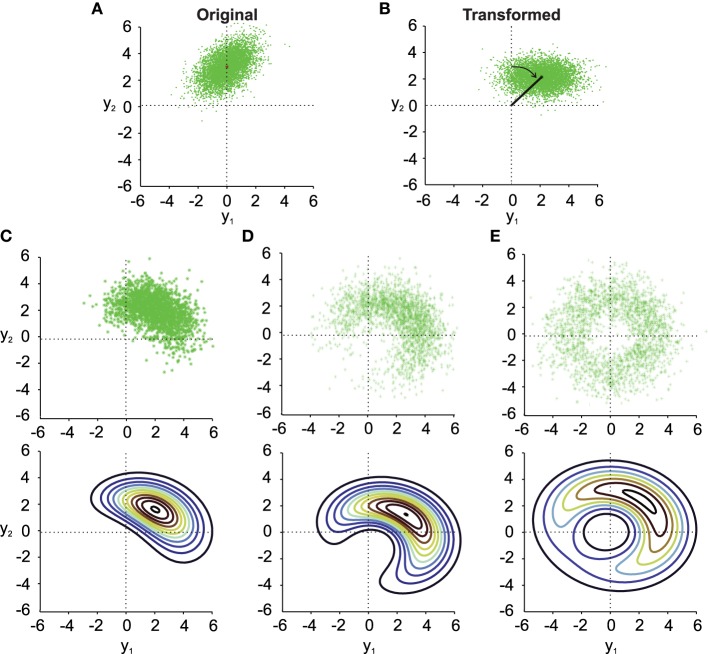
**Noisy rotations. (A)** Scatter plot of *n* = 5000 original normally distributed data points with μ_*x*_1__ = 0, μ_*x*_2__ = 3, ρ = 0.5 and σ_*x*_1__ = σ_*x*_2__ = 1. **(B)** Same data as in **(A)** rotated by a fixed μ_θ_ = −π/4. **(C–E)** (top). Results from noisy rotations of the data in **(A)** by the same average amount as in **(B)** (μ_θ_ = −π/4) but with different rotational standard deviations, i.e., σ_θ_ = 0.4 **(C)**, σ_θ_ = 1 **(D)**, σ_θ_ = π/2 **(E)**. Bottom parts of **(C–E)** show contour plots of the distribution *f*_*y*_(*y*_1_, *y*_2_) (Equation 10).

In this section we investigate the effect of noise in rotation angles on transformed simulated data (see Figure legends for exact simulation parameters) to investigate the statistical data properties before and after a noisy rotation. To do so, we first numerically computed the integral in Equation (10) to find the distribution of the transformed data with the assumption that the angle of rotation is normally distributed (see Figure 1). We then compare this noisy transformation result to the data transformed without noise in the rotation angle. As noted before, this latter distribution is normal for joint normal original data. All transformations considered were performed under the assumption of independent Gaussian noise in the transformation parameters.

Three things can be observed in Figure 1 when comparing the transformed data without noise in the transformation angle (Figure 1B) to data from noisy transformations (Figures 1C–E). First, it is quite obvious that noise added to the transformation will result in noisier transformed data. This will result in larger variances and covariances of the transformed data. Second, even moderate noise can change the covariance of the transformed data (compare Figure 1B and Figure 1C), both in size and orientation. Third, noise in the transformation angle generally distorts data away from multi-variate normality. This distortion is non-trivial in particular for data with non-zero correlation ρ. This is best observed in the contour plots in the lower part of Figures 1C–E. It is thus important to quantify such distortions, which we will do in the following.

**Figure 2:**
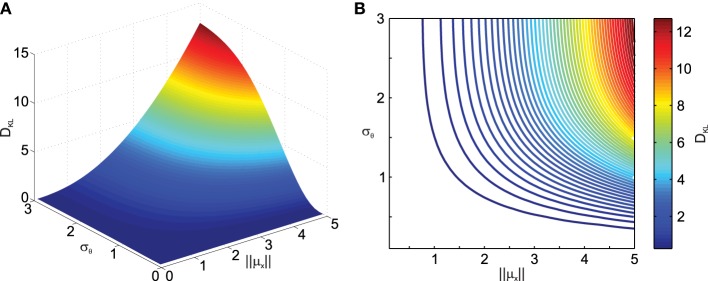
**Effect of transformation angle noise σ_θ_ and data eccentricity from the origin ||μ**_***x***_**|| on the multi-variate normality of the transformed data, as captured by the Kullback–Leibler distance** ***D***_***KL***_ **(Equation 11)**. μ_*x*_1__ = 0, μ_*x*_2__ = 1, ρ = 0.5 and σ_*x*_1__ = σ_*x*_2__ = 1. **(A)** Surface plot. **(B)** Corresponding contour plot.

To obtain a better idea about how noise in transformations affects the distribution of the transformed data, we quantified the difference between the distribution of the data transformed under noisy conditions and the transformed data without noise in the transformation using the Kullback–Leibler distance (*D*_*KL*_) measure defined in Equation (11). Figure 2 shows the result of the deviation from normality analysis. As one can see, *D*_*KL*_ saturates for large transformation angle noise σ_θ_ but grows fast with the data eccentricity from the origin ||μ_*x*_||. The former is observed because with infinite σ_θ_ the data become uniformly distributed on an annulus, while the latter occurs because for small ||μ_*x*_|| the original data distribution, i.e., the variability ellipse, spans the origin and thus transformation noise does not have a big impact. It should also be noted that *D*_*KL*_ is invariant to the mean transformation angle ||μ_θ_|| (data not shown).

**Figure 3:**
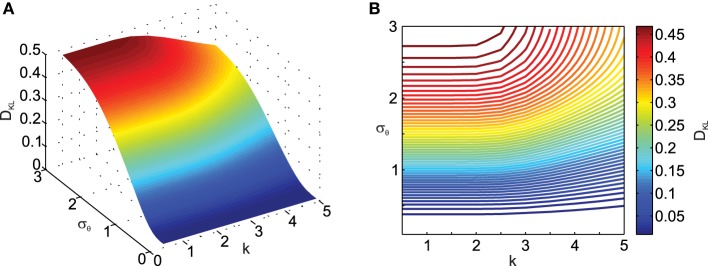
**Effect of transformation angle noise σ_θ_ and scaling factor** ***k*** **on the multi-variate normality of the transformed data, as captured by the Kullback–Leibler distance** ***D***_***KL***_ **(Equation 11)**. μ_*x*_1__ = 0, μ_*x*_2__ = 1, ρ = 0.5 and σ_*x*_1__ = σ_*x*_2__ = 1. **(A)** Surface plot. **(B)** Corresponding contour plot.

Next we analyzed the effect of the scaling factor *k* on *D*_*KL*_, also as a function of σ_θ_ in Figure 3. Here one can observe the effect of σ_θ_ on *D*_*KL*_ for small ||μ_*x*_|| (i.e., ||μ_*x*_|| = 1), which was not visible in Figure 2 due to effect scaling. Interestingly, *k* only has an influence on *D*_*KL*_ for large σ_θ_, actually reducing the deviation from normality. This is because scaling in the transformation will result in data being pushed away from the origin and thus result in smaller relative deviations from normality.

**Figure 4:**
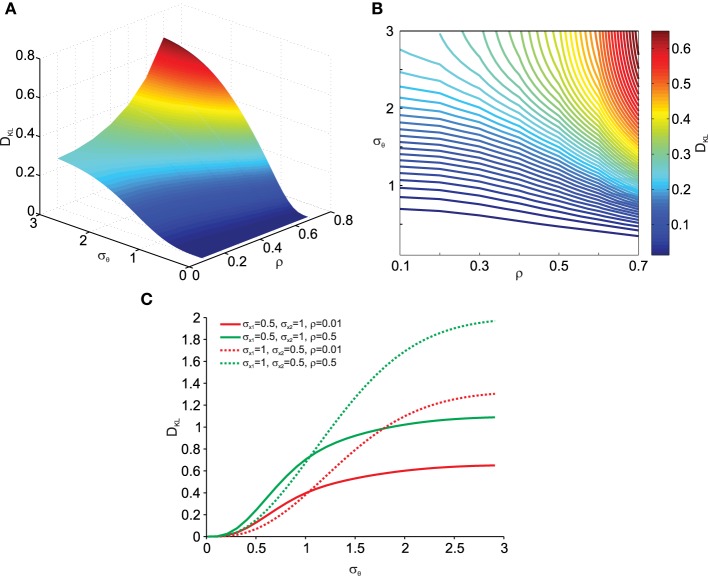
**Effect of transformation angle noise σ_θ_ and data correlation ρ on the multi-variate normality of the transformed data, as captured by the Kullback–Leibler distance** ***D***_***KL***_ **(Equation 11)**. μ_*x*_1__ = 0, μ_*x*_2__ = 1 and σ_*x*_1__ = σ_*x*_2__ = 1. **(A)** Surface plot. **(B)** Corresponding contour plot. **(C)** Effect of transformation angle noise σ_θ_ on *D*_*KL*_ for different combinations of original data variances (σ_*x*_1__ and σ_*x*_2__) and correlations ρ.

More interestingly we also analyzed the effect of data correlation ρ and σ_θ_ on *D*_*KL*_. This is shown in Figures 4A, B. As can be observed, deviations from normality grow with increasing data correlation ρ. Thus, increased covariances (and variances) in the data make the transformation result more vulnerable to noise effects. In addition, Figure 4C shows that this relationship depends on the relative contribution of σ_*x*_1__ and σ_*x*_2__. Thus the orientation of the correlated noisy original data with respect to the eccentricity (μ_*x*_1__ and μ_*x*_2__) from the origin is an important factor in how noise in the rotation angle influences multi-variate normality.

While deviations from normality can be quantitatively assessed when both the original and transformed data are available, this is not usually the case when dealing with experimental measurements where we often do not have access to the original data distribution but only measure the data after it has been transformed. In order to still be able to assess deviations from normality and to do so regardless of data dimensionality, we developed a novel measure based on the sample's Mahalanobis distance (see Equation 12). This has three advantages. First, it reduces the multi-dimensional data to a one-dimensional measure; second, the Mahalanobis distance (by definition) normalizes the deviations from the mean by the variance thus providing a scale-invariant measure; and three, the Mahalanobis distance of normally distributed data follows a χ^2^ distribution. The latter means that we can generate χ^2^-distributed data for comparison with experimental data if the original data is not available.

**Figure 5:**
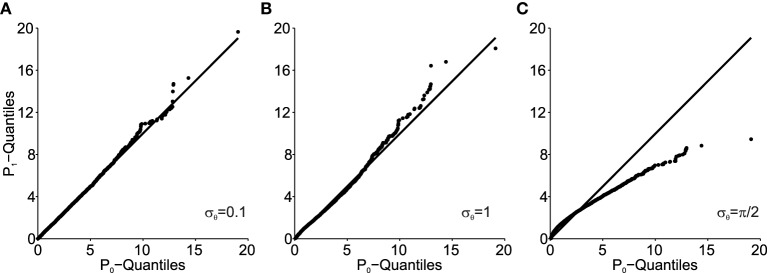
**Q–Q plots of the transformed data (*****P*****_1_) Mahalanobis distance against the original data (*****P*****_0_) for different values of angular transformation noise σ_θ_. (A)** σ_θ_ = 0.1. The Q–Q plot compares an ordered sample distribution with the quantiles of the standard normal distribution. **(B)** σ_θ_ = 1. **(C)** σ_θ_ = π/2. Deviations from the unity line indicate deviations from normality.

Figure 5 shows how the Mahalanobis distance of the transformed data behaves as a function of angular transformation noise σ_θ_ when plotted as Q–Q plots. As shown, the larger σ_θ_, the more the data deviates from the unity line. The unity line represents equal *P*_1_ and *P*_0_ distributions and thus the larger the deviations from the unity line, the larger the deviation from normality of *P*_1_ (note, that original data *P*_0_ is normally distributed here).

**Figure 6:**
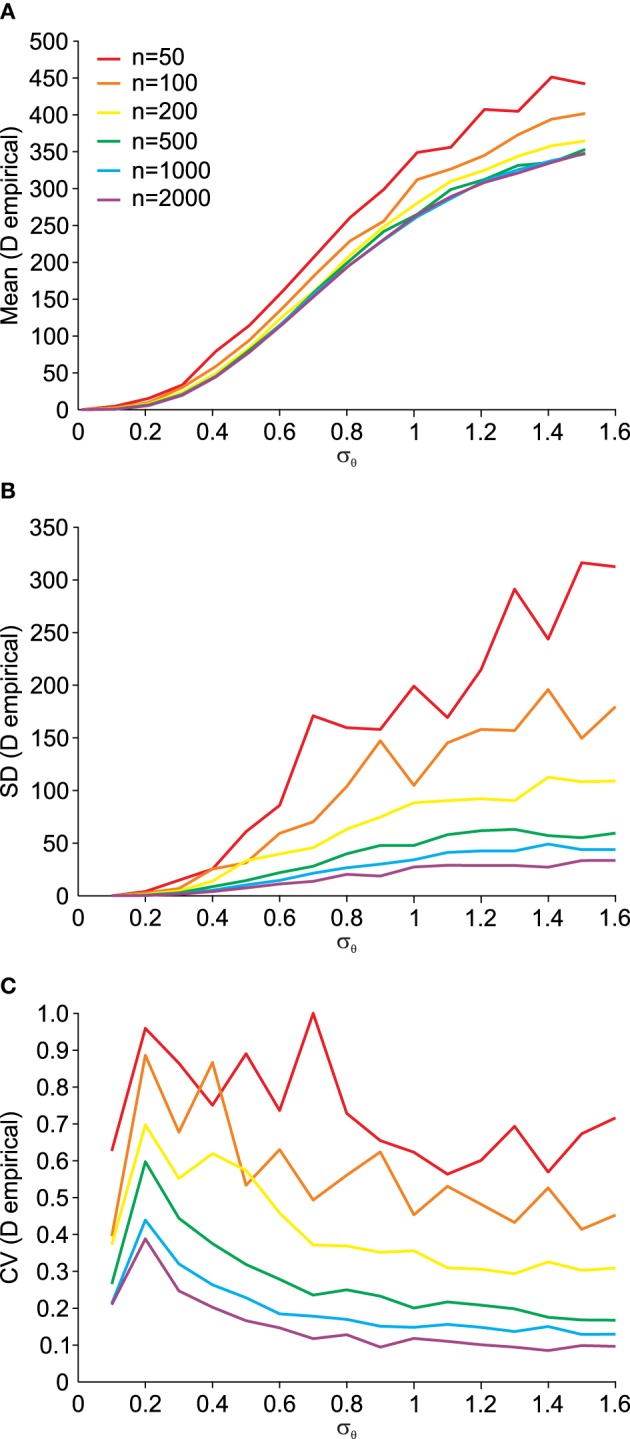
**Dependency of the empirical distance** ***D*** **from multi-variate normality (see Equation 14) on the angular transformation noise σ_θ_ and the number of available data points** ***n*****. (A)** Average (Mean) empirical distance *D*. **(B)** Standard deviation (SD) of empirical distance *D*. **(C)** Coefficient of variation (CV = SD/Mean) of empirical distance *D*.

Using the procedure illustrated by the Q–Q plots (Figure 5), we can quantify the deviation from normality in a single measure, as outlined in Equation (14). Using this single measure of the empirical distance *D* from normality, we can analyze its susceptibility to data set size *n*. This is done in Figure 6. The small influence from data set size on the mean empirical distance *D* (Figure 6A) stems from the random nature of the samples and *P*_0_ and *P*_1_ here being independently generated, i.e., *P*_1_ is not a rotated version of *P*_0_. We did this to analyze the usefulness of this empirical measure for real data where original distributions are often not available and have to be created based on an assumption of the underlying distribution. Thus, for small effects and limited data it is preferable to only compare *D* across data sets of equal size. As expected, the variance of *D* also depends on the data set size *n* (Figure 6B) and as a result so does the coefficient of variation (Figure 6C). The larger the data set size *n*, the more robust the estimation of *D*.

**Figure 7:**
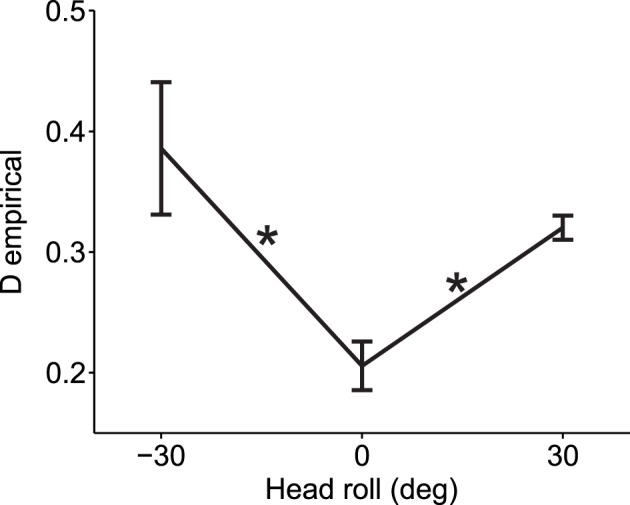
**Experimental validation of deviation from normality measure**. When the head was rolled and thus a larger reference frame transformation was needed, data deviated more from the normal distribution as compared to when the head was straight (head roll = 0). Average measures across all 7 subjects and across all six reach targets are shown for each head roll angle (means ± s.e.m.). Asterisks indicate significant differences (ANOVA with *post-hoc* paired *t*-tests, *p* < 0.05). Data points at −30 and 30° head roll angle were not significantly different from one another (*p* > 0.1).

To demonstrate that our empirical measure of deviations from normality can be effectively used for real data, we computed the deviation from normality for reaching movements under different head roll angles as published in Burns and Blohm, 2010 (see Methods for more details). For visually-guided reaches during head roll, the brain has to transform rotated visual inputs into spatially accurate reach motor commands. This requires a reference frame transformation. Burn and Blohm (2010) have shown in accordance with other studies (Sober and Sabes, 2003, 2005; McGuire and Sabes, 2009; Burns et al., 2011) that such reference frame transformations introduce noise, and that the larger the transformation angle, the more noise is added. Based on our SRFT theory, this should lead to changes to the normality of reach distributions. Specifically, we expect larger deviations from normality when the head is rolled as opposed to when the head is upright. We test this hypothesis in Figure 7. As one can observe, data is deviated from normality even when the head is upright. This might have many causes, including measurement errors, biomechanical factors or workspace anisotropies (e.g., we use our right arm more for rightward reaches). Regardless, the important observation is to compare deviations from normality when the head is rolled to when the head is upright. Doing so in Figure 7, we find that reaching under eccentric head rolls leads to significantly larger deviations from normality than reaching when the head is upright [Two-Way ANOVA with factors subjects and head roll; main effect of head roll *F*_(2, 117)_ = 6.27, *p* = 0.0026; main effect of subjects *F*_(6, 117)_ = 0.96, *p* = 0.45; no interaction effect). This validates our hypothesis and confirms that reference frame transformations in the brain should indeed be viewed as being stochastic in nature.

## 4. Discussion

We have studied the impact of stochastic noise on reference frame transformations and argue that SRFTs can lead to distortions of the statistical distribution of transformed data. In neuroscience, this idea has been previously suggested (Rossetti et al., 1994; Sober and Sabes, 2003, 2005; Blohm and Crawford, 2007; Schlicht and Schrater, 2007; McGuire and Sabes, 2009; Burns and Blohm, 2010; Burns et al., 2011; Tagliabue and McIntyre, 2011, 2013, 2014) but never formally quantified. Indeed, noise added in reference frame transformations should lead to larger variability (in terms of variance) in the system's output. This has been reported for the geometry-dependence of hand localization (Scott and Loeb, 1994; Fuentes and Bastian, 2010), for reaching movements (Rossetti et al., 1994; Blohm and Crawford, 2007; Schlicht and Schrater, 2007; Burns and Blohm, 2010) and for sensory-motor transformations requiring explicit reference frame transformation (Blohm and Crawford, 2007; Burns and Blohm, 2010). We propose a new method to capture the changes of the statistical distribution of experimental data after a reference frame transformation, which tells us that the sensory-to-motor transformation in the brain involves a SRFT based on noisy estimates of the head roll angle (Steinleitner, 1978; Guerraz et al., 2000).

Reference frame transformations are omnipresent in the brain (see Introduction). Therefore we argue that quantifying those SRFTs could provide deep insight into the working principles of the brain. This includes research areas as diverse as multi-sensory integration across reference frames, coordinate transformations in sensory-motor planning and forward/inverse models in motor control. Indeed, based on our theory, one would expect deviations from specific distributions (usually Gaussian) in many of these studies. Our framework will for the first time allow quantifying these effects. In addition, many other research areas face similar problems. For example, in the pose estimation industry different sensors capture data in different reference frames that need to be combined for a unique pose estimate and the relative orientation of those reference frames is often not fixed (such as for a sensor attached to the moving body). In that case, the relative orientation between reference frames needs to be estimated from (noisy) sensory data. Thus SRFTs should be used to quantify the reliability of individual sensory information in the generation of the unique pose estimate. Thus we believe that this study has broad implications for science and industry that go beyond neuroscience research.

The method presented herein can be used to quantify how different experimental conditions affect output statistics, and thus to indirectly estimate the degree of stochasticity of the SRFTs involved, which can provide insight into different processing steps in the brain. There are of course limitations to our framework. So far we have explored 2D effects and did not consider higher dimensions. In general, only translational transformations will maintain normality, which scaling or rotations can result in deviation from the original distribution. It is straight-forward to extend our main findings to 3D rotations, as data can be projected onto a 2D plane orthogonal to the rotation axis. However, investigating the effect of uncertainty in the orientation of the rotation axis remains to be done. Another limitation is that the original data (before any reference frame transformation) is often not known and has to be assumed, at least in neuroscience research. This also means that we do not know what the ideal distribution shape should be, as required by the empirical distance measure. However, one can get around this problem by simply assuming a certain distribution and computing deviations from that distribution for different conditions, such as we have done here. Overall, these limitations are easily overcome in practice and should not prevent successful application of our theory.

## Funding

This study was supported by NSERC (Canada), CFI (Canada) and ORF (Canada).

### Conflict of interest statement

The authors declare that the research was conducted in the absence of any commercial or financial relationships that could be construed as a potential conflict of interest.

## References

[B1] Blohm G. Crawford J. D. (2007). Computations for geometrically accurate visually guided reaching in 3-d space. J. Vis. 7, 1–22. 10.1167/7.5.4 18217844

[B2] Buneo C. A. Andersen R. A. (2006). The posterior parietal cortex: sensorimotor interface for the planning and online control of visually guided movements. Neuropsychologia 44, 2594–2606. 10.1016/j.neuropsychologia.2005.10.011 16300804

[B3] Burdenski T. K. (2000). Evaluating univariate, bivariate, and multivariate normality using graphical procedures. Mult. Lin. Regression Viewpoints 26, 15–28.

[B4] Burns J. K. Blohm G. (2010). Multi-sensory weights depend on contextual noise in reference frame transformations. Front. Hum. Neurosci. 4:221. 10.3389/fnhum.2010.00221 21165177 PMC3002464

[B5] Burns J. K. Nashed J. Y. Blohm G. (2011). Head roll influences perceived hand position. J. Vis. 11:3. 10.1167/11.9.3 21824979

[B6] Cohen Y. E. Andersen R. A. (2002). A common reference frame for movement plans in the posterior parietal cortex. Nat. Rev. Neurosci. 3, 553–562. 10.1038/nrn873 12094211

[B7] Crawford J. D. Medendorp W. P. Marotta J. J. (2004). Spatial transformations for eye-hand coordination. J. Neurophysiol. 92, 10–19. 10.1152/jn.00117.2004 15212434

[B8] Engel K. C. Flanders M. Soechting J. F. (2002). Oculocentric frames of reference for limb movement. Arch. Ital. Biol. 140, 211–219. 12173524

[B9] Fuentes C. T. Bastian A. J. (2010). Where is your arm? Variations in proprioception across space and tasks. J. Neurophysiol. 103, 164–171. 10.1152/jn.00494.2009 19864441 PMC4116392

[B10] Guerraz M. Luyat M. Poquin D. Ohlmann T. (2000). The role of neck afferents in subjective orientation in the visual and tactile sensory modalities. Acta Otolaryngol. 120, 735–738. 10.1080/000164800750000261 11099150

[B11] Henderson A. R. (2006). Testing experimental data for univariate normality. Clin. Chim. Acta Int. J. Clin. Chem. 366, 112–129. 10.1016/j.cca.2005.11.007 16388793

[B12] Henriques D. Y. Medendorp W. P. Khan A. Z. Crawford J. D. (2002). Visuomotor transformations for eye-hand coordination. Prog. Brain Res. 140, 329–340. 10.1016/s0079-6123(02)40060-x 12508600

[B13] Knudsen E. I. Lac S. Esterly S. D. (1987). Computational maps in the brain. Annu. Rev. Neurosci. 10, 41–65. 10.1146/annurev.ne.10.030187.000353 3551761

[B14] Kullback S. Leibler R. A. (1951). On information and sufficiency. Ann. Math. Stat. 22, 79–86. 10.1214/aoms/1177729694

[B15] Lacquaniti F. Caminiti R. (1998). Visuo-motor transformations for arm reaching. Eur. J. Neurosci. 10, 195–203. 10.1046/j.1460-9568.1998.00040.x 9753127

[B16] McGuire L. M. Sabes P. N. (2009). Sensory transformations and the use of multiple reference frames for reach planning. Nat. Neurosci. 12, 1056–1061. 10.1038/nn.2357 19597495 PMC2749235

[B17] Rossetti Y. Tadary B. Prablanc C. (1994). Optimal contributions of head and eye positions to spatial accuracy in man tested by visually directed pointing. Exp. Brain Res. 97, 487–496. 10.1007/BF00241543 8187860

[B18] Schlicht E. J. Schrater P. R. (2007). Impact of coordinate transformation uncertainty on human sensorimotor control. J. Neurophysiol. 97, 4203–4214. 10.1152/jn.00160.2007 17409174

[B19] Scott S. H. Loeb G. E. (1994). The computation of position sense from spindles in mono- and multiarticular muscles. J. Neurosci. 14, 7529–7540. 7996193 10.1523/JNEUROSCI.14-12-07529.1994 PMC6576884

[B20] Snyder L. H. (2000). Coordinate transformations for eye and arm movements in the brain. Curr. Opin. Neurobiol. 10, 747–754. 10.1016/S0959-4388(00)00152-5 11240284

[B21] Sober S. J. Sabes P. N. (2003). Multisensory integration during motor planning. J. Neurosci. 23, 6982–6992. 12904459 10.1523/JNEUROSCI.23-18-06982.2003 PMC6740676

[B22] Sober S. J. Sabes P. N. (2005). Flexible strategies for sensory integration during motor planning. Nat. Neurosci. 8, 490–497. 10.1038/nn1427 15793578 PMC2538489

[B23] Soechting J. F. Flanders M. (1992). Moving in three-dimensional space: frames of reference, vectors, and coordinate systems. Annu. Rev. Neurosci. 15, 167–191. 10.1146/annurev.ne.15.030192.001123 1575441

[B24] Steinleitner S. L. (1978). Interaction of labyrinthine and somatoreceptor inputs as determinants of the subjective vertical. Psychol. Res. 40, 65–76. 10.1007/BF00308464 635075

[B25] Tagliabue M. McIntyre J. (2011). Necessity is the mother of invention: reconstructing missing sensory information in multiple, concurrent reference frames for eyeï¿½hand coordination. J. Neurosci. 31, 1397–1409. 10.1523/JNEUROSCI.0623-10.2011 21273424 PMC6623599

[B26] Tagliabue M. McIntyre J. (2013). When kinesthesia becomes visual: a theoretical justification for executing motor tasks in visual space. PLoS ONE 8:e68438. 10.1371/journal.pone.0068438 23861903 PMC3702599

[B27] Tagliabue M. McIntyre J. (2014). A modular theory of multisensory integration for motor control. Front. Comput. Neurosci. 8:1. 10.3389/fncom.2014.00001 24550816 PMC3908447

[B28] Thompson B. (1990). Multinor: a fortran program that assists in evaluating multivariate normality. Educ. Psychol. Meas. 50, 845–848. 10.1177/0013164490504014

[B29] Wilk M. B. Gnanadesikan R. (1968). Probability plotting methods for the analysis of data. Biometrika 55, 1–17. 10.2307/2334448 5661047

